# Leave Me Alone With Your Symptoms! Social Exclusion at the Workplace Mediates the Relationship of Employee's Mental Illness and Sick Leave

**DOI:** 10.3389/fpubh.2022.892174

**Published:** 2022-07-28

**Authors:** Benjamin Pascal Frank, Clara Magdalena Theil, Nathalie Brill, Hanna Christiansen, Christina Schwenck, Meinhard Kieser, Corinna Reck, Ricarda Steinmayr, Linda Wirthwein, Kathleen Otto, The COMPARE-family Research Group, Kristin Gilbert

**Affiliations:** ^1^Department of Work and Organizational Psychology, Faculty of Psychology, Philipps University Marburg, Marburg, Germany; ^2^Department of Clinical Child and Adolescent Psychology, Faculty of Psychology, Philipps University Marburg, Marburg, Germany; ^3^Department of Special Needs Educational and Clinical Child and Adolescent Psychology, Faculty of Psychology and Sports Science, Justus-Liebig-University Giessen, Giessen, Germany; ^4^Department of Medical Biometry, Institute of Medical Biometry and Informatics, Heidelberg University, Heidelberg, Germany; ^5^Department of Clinical Child and Adolescent Psychology, Faculty of Psychology, Ludwig-Maximilians-University München, Munich, Germany; ^6^Department of Educational Psychology, Faculty of Education, Psychology, and Sociology, Institute of Psychology, Technical University Dortmund, Dortmund, Germany

**Keywords:** workplace, mental illness, social exclusion, sick leave, discrimination, stereotype content model

## Abstract

Although a substantial part of employees suffers from a mental illness, the work situation of this population still is understudied. Previous research suggests that people with a mental illness experience discrimination in the workplace, which is known to have detrimental effects on health. Building on the stereotype content model and allostatic load theory, the present study investigated whether employees with a mental illness become socially excluded at the workplace and therefore show more days of sick leave. Overall, 86 employees diagnosed with a mental disorder were interviewed and completed online-surveys. Path analyses supported the hypotheses, yielding a serial mediation: The interview-rated severity of the mental disorder had an indirect effect on the days of sick leave, mediated by the symptomatic burden and the social exclusion at the workplace. In the light of the costs associated with absenteeism the present paper highlights the harmfulness of discrimination. Organizations and especially supervisors need to be attentive for signs of exclusion within their teams and try to counteract as early as possible.

## Introduction

Mental illnesses are one of the main causes of disability worldwide (1, 2). Estimates indicate that more than one in six people across the European countries (17.3%) experienced mental health problems in 2016 (3). Besides severe cognitive, emotional, and behavioral impairments for the affected individuals (4–6), mental health problems pose a substantial economic burden on health care systems (7, 8). Since a substantial part of the working population suffers from mental disorders (9, 10), mental illnesses furthermore interfere with the functioning of employees and organizations as well: Several studies provide evidence for a significant relationship between employees' mental health status and their performance (11–13). Thus, mental disorders contribute to a substantial amount of indirect costs organizations spend, arising from reduced productivity or increased absenteeism of their employees with a mental illness (14, 15).

Despite these consequences of mental disorders on the well-being of employees and organizations, the work situation of employees with a mental illness (EMI) still is understudied (16). Investigations suggest that EMI are faced with various barriers at their jobs (17, 18), which in turn could worsen their health status (19). Stigmatization and discrimination of people with a mental illness for instance are not only common in the general public (20, 21), but also appear in organizations, for instance in form of the exclusion from work-related events or the denial of conversation in general (22–24). Discriminative actions as this social exclusion are known to be detrimental for health (25–29) and thus social exclusion might result in increased absenteeism (30). However, to the best of our knowledge, the influence of the mental illness on social exclusion and absenteeism has not been investigated jointly yet. This study is intended to close this research gap and thereby to contribute to a better understanding of the negative effects mental disorders and social exclusion have on employees and organizations, hoping to provide starting points for their mitigation.

In a recent review, Follmer and Jones (16) not only highlight the existence of negative stereotypes about EMI among supervisors and employees, but also the discrimination they experience in the workplace. An explanation why and how such negative stereotypes about people with a mental disorder end up in discriminative behavior provides the stereotype content model (SCM) (31) and its extension, the behaviors from intergroup affect and stereotypes (BIAS) map (32). The SCM proposes that two dimensions are central for the emergence of different group stereotypes: *warmth*, defined as the perception whether the intent of a certain group toward one self or one's ingroup is either beneficial or malevolent and *competence*, defined as the perceived capability of the group to pursue and enact those intentions (31, 33). Groups are classified on both dimensions, yielding either thoroughly positive stereotypes (i.e.„ classified as warm and competent) as e.g., the middle class, thoroughly negative stereotypes (i.e., cold and incompetent) as e.g., poor people, or ambivalent stereotypes (i.e., warm and incompetent or cold and competent) as e.g., elderly or rich people (32, 33). Each of those stereotype-combinations elicits specific emotional reactions toward the classified group (31, 33): Warm and competent groups are admired, cold and incompetent groups elicit contempt, warm but incompetent groups induce pity, and cold but competent groups envy. Those emotional reactions finally end up in specific behavioral tendencies toward the classified group, mediating the effect of the stereotype on the behavior as the BIAS map proposes (32, 33): While admired groups induce active and passive facilitation, the opposite is true for resented groups (i.e., incompetent and cold) which provoke active and passive harm. Envied or pitied groups evoke mixed behavioral patterns (passive facilitation and active harm or active facilitation and passive harm, respectively). A multiplicity of studies investigated the SCM and the BIAS map and found support for their assumptions, also across various nations, including Germany [e.g., (31, 32, 34–36)].

Hence, according to the SCM, emotional reactions and behavioral tendencies toward people with a mental illness depend on the perception whether (a) they want to help or harm oneself and whether (b) they are able to do so. In a systematic literature review, Parcesepe and Cabassa (21) sum up that people with a mental illness are often perceived as being e.g., incompetent, dangerous and criminal, indicating a rather low evaluation on the two dimensions warmth and competence. In a more proximal study on the SCM, Sadler et al. (37) asked participants to rate the warmth and competence of people with a mental disorder as seen by Americans in general: Results confirmed the indirect evidence, showing that people with a mental illness are perceived as equally incompetent and cold as poor people. Accordingly, people tend to react with active harm, e.g., segregation (38, 39), or passive harm, e.g., social distance (20, 21, 38), toward them, as predicted by the SCM.

Although research on employees with a mental illness in general still is scarce [see Follmer and Jones (16) for a recent review], the existing literature indicates that likewise discriminatory behaviors toward people with a mental illness also exist in the workplace [e.g., (17, 18, 22, 24, 40, 41)]. Follmer et al. (23) for instance found that lower ratings of warmth and competence of EMI predict a higher desire to socially distance oneself from a fictious coworker with a mental illness. This confirms investigations on the experiences of EMI, reporting that people at work avoid them due to their mental health problems (24). Thus, people with a mental illness do not only experience discriminatory behavior as social exclusion in the general public, but also in their workplaces.

The evaluation that a specific colleague has a mental health problem and the subsequent exclusion of this colleague by the coworkers however, do not appear out of the blue: First, the coworkers have to notice corresponding peculiar behavior (or in other words: symptoms) that marks the colleague accordingly. Only if the colleague acts strangely (i.e., displays symptoms), an appraisal as being (more or less) mentally ill on the part of the coworkers is possible—followed by the above mentioned perception that the colleague is cold and incompetent [c.f. (37)] and the corresponding behavioral reactions (e.g., social exclusion) toward her/him [c.f. (33)]. Thus, EMI with a higher symptomatic burden (i.e., more and/or stronger symptoms) should be perceived as being more mentally ill—and thus experience more social exclusion at the workplace.

The symptomatic burden on the other hand is inevitably linked to the severity of the mental illness itself: The level of disability and distress patients experience in various life domains, including occupation, increases as the mental health status gets more severe (42, 43). More depressed people for instance spend less time in groups, use more negative emotion words, and feel lonely more often than their less depressed counterparts (44, 45). Thus, an increasing severity of the mental illness should go along with more and/or stronger symptoms—or in other words a higher symptomatic burden. This in turn will be noticed by the coworkers, leading to social exclusion of the EMI.

Based on this line of thought we therefore test the following hypotheses:

Hypothesis 1: The severity of the mental disorder leads to a higher symptomatic burden.
Hypothesis 2: The severity of the mental disorder leads to an increase of experienced social exclusion at the workplace via a higher symptomatic burden.

Social stressors as social exclusion are not only uncomfortable to endure but unfortunately also could have detrimental effects on human body and thus health according to the allostatic load model (46–49). The allostatic load model assumes that physiological reactions mediate the effect of (job) stressors on health outcomes (48, 50). According to the model, different physiological systems in the human body initiate an adaptive response if exposed to a stressor, that is the physiological markers increase (48, 49). This response persists until the stressor vanishes (49). Now the physiological response is stopped and the markers decrease – thus recovery takes place (48, 49). This process of adjustment in order to cope with a stressor fulfilled by different physiological systems is defined as *allostasis* (50, 51).

The described switching on and off of the physiological response is an adaptive and beneficial mechanism—however it can become overstrained with potential detrimental effects on the human body in the long run, a status called *allostatic load* (46–49). The allostatic system for instance may have difficulties to habituate to the same stressor (i.e., the physiological response to the stressor is always equally high) or problems to end the response adequately (i.e., it continues even after the stressor disappears)—both resulting in a hyperactivation of the system (48, 49). McEwen (49) calls these subtypes of allostatic load “lack of adaption” and “prolonged response” and further postulates that the effects of a chronic hyperarousal add up over time and finally result in diseases (47–49).

Unfortunately, social stressors are designated to cause those subtypes of allostatic load: Various psychophysiological studies indicate that even when people are exposed to the same social stressor for several times, the sympathetic response does not significantly change, suggesting poor habituation of the associated system to the stressor [e.g., (52–55)]—or (in the sense of allostatic load theory) a “lack of adaption.” Furthermore, employees tend to ruminate in their leisure time when they are confronted with social stressors at work (56–58). Such pondering about a stressor can lead to an extended physiological reaction, as experimental laboratory studies suggest (59)—or in other words can cause a ”prolonged response” in the sense of allostatic load theory. Thus, if people are exposed to the same social stressor over and over again—as it can be the case for employees confronted with social exclusion at the workplace—both mentioned subtypes of allostatic load might occur and finally end up in sickness.

The proposed impact of social exclusion on human health was supported by research from various fields, indicating that isolation in general affects the functioning of the immune system and even mortality rates (27–29). But there also is according evidence in the work environment since socially excluded employees have a higher risk for a long-term sick leave spell (30), just as victims of workplace bullying do [for a recent review and meta-analysis see (60)]. Thus, it is possible that the influence of the mental illness goes beyond the symptoms a patient suffers from and the social exclusion (s)he experiences thereof at the workplace: The isolation could furthermore lead to an increase of days EMI are sick leaving, yielding a serial mediation of the severity of the mental illness on sick leave via the symptomatic burden and social exclusion at the workplace. While the debarment of EMI as well as the impact of social exclusion on absenteeism has already been under examination (23, 24, 30), no study has ever combined those research lines and investigated the influence of the mental illness on sick leave via social exclusion.

Based on this line of thought we therefore test the following hypothesis:

Hypothesis 3: The severity of the mental disorder leads to an increase of sick leave, sequentially via a higher symptomatic burden and more social exclusion at the workplace.

## Materials and Methods

### Design and Procedure

The data of the present study originate from the COMPARE-consortium (61) standing for “children of mentally ill parents at risk evaluation.” The consortium investigates why children of parents with a mental illness are at higher risk of developing mental illnesses themselves and whether a preventive intervention may interrupt this malicious transmission of the parental mental state to the child (61). It consists of a clinical study “COMPARE family” [see (62)] as well as four subprojects named “COMPARE emotion,” “COMPARE interaction,” “COMPARE work” and “COMPARE school” [see (61)]. In the following we only report the aspects of the COMPARE-consortium which are relevant for the paper at hand. For more details on the consortium and the subprojects we refer to Christiansen et al. (61) and for more information on the clinical study to Stracke et al. (62)^1^.

The recruitment for the used partial data set took place from January 2018 to May 2020 in different university outpatient clinics throughout Germany. The clinical study was advertised with e.g., flyers, newspaper articles and information meetings for professionals (e.g., physicians). Persons contacting the clinics asking for psychotherapeutic help were screened for eligibility and interest in participating in the clinical study [c.f. (62)]. After deciding to participate, they signed the informed consent and fulfilled the first assessment, from which the data used in this paper originate.

Every assessment was split in multiple occasions: First, structured interviews were conducted in two sessions on site by trained study personal. After the second interview session, patients received a sheet with a link to the online questionnaire assessed by “COMPARE family” and were asked to answer it at home [c.f. (62)]. If participants also agreed to take part in “COMPARE work” (or one of the other subprojects) they furthermore received links to the corresponding online questionnaires.

Different criteria had to be met for patients to participate in the clinical study: (1) they had to search for outpatient psychotherapeutic care, (2) they had to fulfill the diagnostic criteria for a DSM-5 disorder (63), and (3) they had to care for at least one child aged between 1.5 and 16 years [c.f. (62)]. Patients were not included in the clinical study if (1) the patient already had been in psychotherapeutic treatment at the present time, (2) the patient needed an acute inpatient treatment, (3) all children fulfilled the criteria for a severe mental illness and furthermore were in need of an immediate treatment, (4) the patient used benzodiazepines regularly (an intermittent use less than once every 2 weeks was allowed) or (5) the family had insufficient German language skills [c.f. (62)]. For the present paper we furthermore excluded patients without a current (self-employment) as well as patients being on a sick leave for longer than 6 weeks since any item with regard to the current work situation could presumably not have been reasonably answered.

### Measures

All data used in this paper have been collected and managed with REDCap (64), standing for “research electronic data capture”. As already mentioned, the data—although belonging to one assessment—were partly measured at different events. Thus, in addition to describing the variables used in the present paper we also mention the occasion they were collected in.

### Variables Assessed in Interview Sessions

#### Severity of Mental Illness

The Diagnostic Interview for Mental Disorders (DIPS) (65, 66) was conducted with the patients in the first interview session. Trained study personnel executed the DIPS [c.f. (62)] taking between 60 and 120 min [c.f. (65)]. The structured interviews of the DIPS-family are a reliable and valid method for diagnosing mental disorders across the lifespan (65). At the end of the interview, the assessor rates the severity of the main diagnosis on a scale from 0 to 8 with digits between 0 and 3 standing for a subclinical diagnosis and digits between 4 and 8 for a clinical diagnosis [c.f. (65, 66)]. This rating was used as an indicator for the severity of the patients' mental illness in the present study. Since subclinical diagnoses were not sufficient for being enrolled into the clinical study, the rating only varied between 4 and 8 in the present paper.

### Variables Assessed in the Questionnaire of “COMPARE Family”

#### Symptomatic Burden

The Brief Symptom Inventory (67) was used to assess the symptomatic burden of the patient. The inventory is a short form of the Symptom Check-List-90-R (68) and measures the subjective impairment caused by the symptoms of the patient (67). Various studies indicate the reliability and validity of the inventory, especially if the psychopathology is under study (67, 69). The 53 items ask the participant how much (s)he suffered from different symptoms within the last seven days as, for example, the symptom of “feeling no interest in things” (Cronbach's α = 0.96). Answers ranged from 0 (*not at all*) to 4 (*very much*) on a Likert-scale with labeled intermediate steps.

### Variables Assessed in the Questionnaire of “COMPARE Work”

#### Social Exclusion

We measured social exclusion with the corresponding scale from Zapf and Holz (70) which is an adaption and further development of Frese's and Zapf (71) scale and has proven to be reliable and valid (71, 72). It consists of four items as “you are being ignored and excluded” (Cronbach's α = 0.82). Answers ranged from 1 (*does not apply at all*) to 6 (*completely applies*) on a Likert scale with labeled intermediate steps.

### Sick Leave

Patients indicated the number of days they were on sick leave by answering the following question: “On how many days have you been absent from work for health reasons in the last 4 weeks (without taking into account the days on which you were missing due to an illness of your child / children)?”

### Statistics

To test hypothesis one and two—which stated that patients with a more severe mental illness suffer from more and/or stronger symptoms (H1), and as a result become socially excluded at work more often, yielding an indirect effect (H2)—we conducted a path analysis using the PROCESS macro version 3.4.1 (73) in IBM SPSS Statistics 26 executing Model 4 (simple mediation): First, we regressed the symptomatic burden on the severity of mental illness. Second, we regressed social exclusion on the severity of mental illness and the symptomatic burden simultaneously. Third, we calculated the indirect effect of the severity of the mental illness on social exclusion via the symptomatic burden. Lastly, we regressed social exclusion solely on the severity of mental illness to test for the total effect of the predictor.

To test whether the social exclusion of the patients at work finally results in an increase of absenteeism—yielding a serial mediation with two mediators (H3)—we further conducted a path analysis using Model 6 (serial mediation, two mediators) of the Process macro (73): After rehearsing the first two steps just described we further regressed sick leave on the severity of mental illness, the symptomatic burden and social exclusion simultaneously. Then, we calculated the indirect effect of the severity of mental illness on sick leave via the symptomatic burden and social exclusion. Finally, we regressed sick leave on the severity of mental illness alone to again obtain the total effect model. The significance level (α) was 0.05 for every hypothesis.

As recommended by Hayes (73) the confidence intervals of any indirect effect presented in this paper are percentile bootstrap confidence intervals calculated on the basis of 10,000 bootstrap-samples. We do not report *p*-values of indirect effects since the normal theory approach used for their calculation has different statistical drawbacks and therefore cannot be recommended [e.g., (73)].

## Results

Overall, *N* = 86 patients met the inclusion criteria and answered the questionnaires from “COMPARE work” and “COMPARE family.” Details of the sample can be found in Table 1.

**Table 1:** Sample details.

**Variable**	***M***	***SD***	**%**
**Gender**			
Female			77.90
Male			22.10
**Age (years)**	39.42	6.90	
**Working hours per week**	29.77	11.15	
**Tenure (years)**	7.45	7.31	
**Contract**			
Permanent			84.50
Fixed-term			15.50
**Education**			
Qualification for university entrance			40.50
Qualification for university of applied science entrance			21.40
General Certificate of Secondary Education			31.00
Certificate of Secondary Education			6.00
Other			1.20
Primary diagnosis			
Affective disorders			46.51
Neurotic, stress-related and somatoform disorders			46.51
Schizophrenia, schizotypal and delusional disorders			2.33
Disorders of adult personality and behavior			2.33
Behavioral syndromes associated with physiological disturbances and physical factors			1.16
Behavioral and emotional disorders with onset usually occurring in childhood and adolescence			1.16
**Additional comorbid diagnoses**			63.10

*Primary diagnosis refers to the German version of the ICD 10 (74). n = 84–86*.

### Preliminary Analysis

Means, standard deviations and correlations between the investigated variables are presented in Table 2. As expected, the severity of mental illness had a positive relation with the symptomatic burden of the patient, which in turn correlated positively with social exclusion. Furthermore, sick leave was positively correlated with social exclusion. Thus, the bivariate relations showed the expected pattern.

**Table 2:** Means, standard deviations, and correlations between investigated constructs.

**Variable**	***M***	***SD***	**1**	**2**	**3**	**4**
1. Severity of mental illness	5.98	0.97	–			
2. Symptomatic burden	0.83	0.55	0.32^**^ [0.11, 0.50]	–		
3. Social exclusion	1.43	0.77	−0.02 [−0.23, 0.20]	0.39^***^ [0.19, 0.56]	–	
4. Sick leave	2.49	6.05	−0.02 [−0.23, 0.19]	0.04 [−0.18, 0.26]	0.29^**^ [0.09, 0.48]	–

*Pearson correlation coefficient with 95%-confidence interval in square brackets. Two-sided testing of significance. n = 82–86*.

** *p < 0. 01*,

*** *p < 0.001*.

### Test of Hypotheses

**Figure 1:**
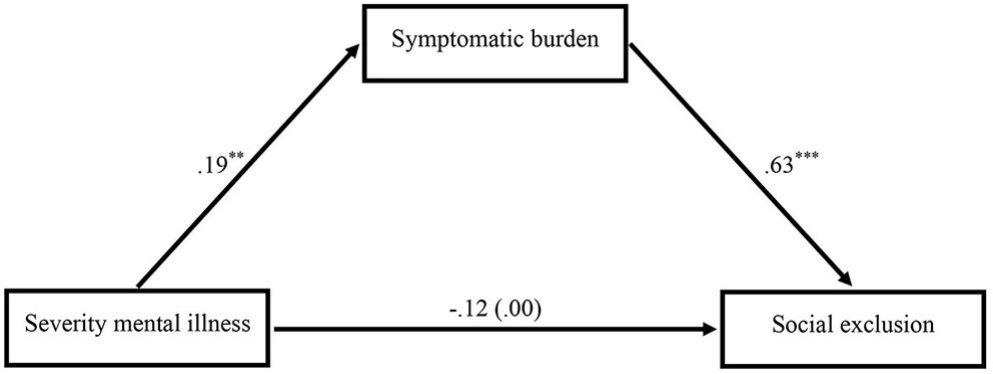
Mediation model showing the impact of the severity of the mental illness on social exclusion via the symptomatic burden (*n* = 82). The total effect of the severity of the mental illness on social exclusion without controlling for the symptomatic burden is shown in parentheses. Unstandardized regression coefficients. Two-sided testing of significance. **p < 0. 01, ***p < 0.001.

The results of our first path analysis which tested the simple mediation model are presented in Figure 1: Patients with a more severe mental illness suffered from more and/or stronger symptoms (*b* = 0.186, *p* = 0.003, 95% CI [0.064, 0.307]) supporting hypothesis 1. Furthermore, employees with a stronger symptomatic burden reported more experienced social exclusion (*b* = 0.628, *p* < 0.001, 95% CI [0.321, 0.935]). There was no direct effect of the severity of the mental illness on social exclusion – neither with (*b* = −0.117, *p* = 0.191, 95% CI [−0.294, 0.060]) nor without (*b* = −0.001, *p* = 0.995, 95% CI [−0.184, 0.182]) controlling for the symptomatic burden. However, there was an indirect effect: Patients with a more severe mental disorder suffered from a stronger symptomatic burden and in turn reported more experienced social exclusion at work (*b* = 0.117, 95% CI [0.028, 0.251]), supporting hypothesis 2.

**Figure 2:**
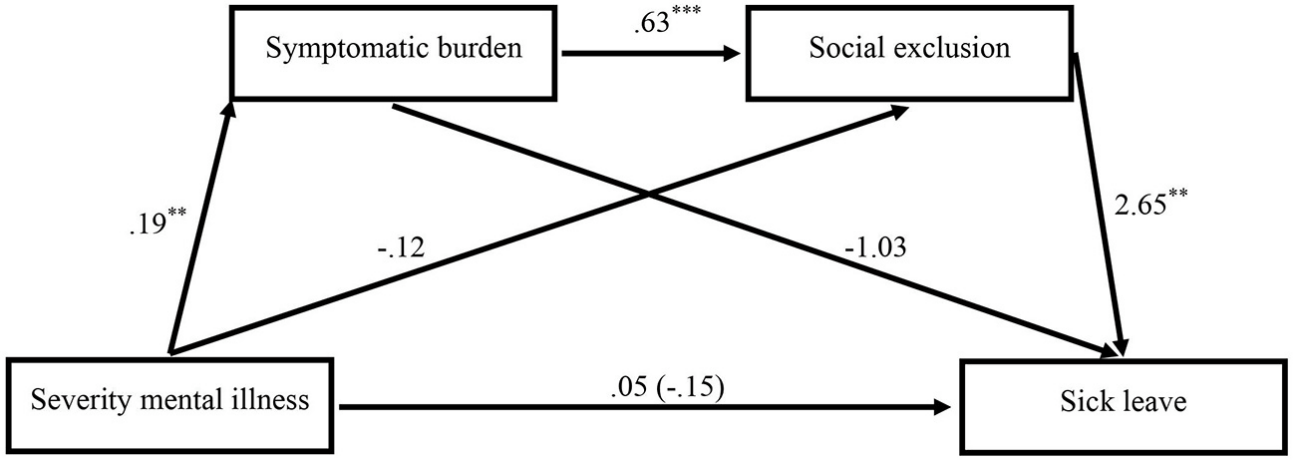
Mediation model showing the impact of the severity of the mental illness on sick leave via the symptomatic burden and social exclusion (*n* = 82). The total effect of the severity of the mental illness on sick leave without controlling for the symptomatic burden and social exclusion is shown in parentheses. Unstandardized regression coefficients. Two-sided testing of significance. **p < 0. 01, ***p < 0.001.

Figure 2 presents the results of the second path analysis which tested the serial mediation model. In addition to Figure 1, it shows that patients who reported more experienced social exclusion were absent from work more often (*b* = 2.652, *p* = 0.006, 95% CI [0.800, 4.504]). Neither the symptomatic burden (*b* = −1.034, *p* = 0.464, 95% CI [−3.829, 1.761]) nor the degree of the mental illness (no matter whether directly (*b* = 0.048, *p* = 0.948, 95% CI [−1.431, 1.528]) or in total (*b* = −0.145, *p* = 0.842, 95% CI [−1.584, 1.294])) were associated with the amount of days a patient was sick leaving at work. Instead, as can be seen in Table 3, the severity of the mental illness had an indirect effect on sick leave: Patients with a more severe mental disorder suffered from a stronger symptomatic burden and reported in turn more experienced social exclusion at work which went along with an increase of days a patient was sick leaving (*b* = 0.309, 95% CI [.022, 0.777]), supporting hypothesis 3. The indirect effects of the severity of the mental illness on absenteeism via the symptomatic burden or social exclusion reclusively were both statistically negligible (see Table 3).

**Table 3:** Indirect effects of the severity of the mental illness on sick leave.

**Effects**	***b***	***SE_***b***_***	**95% CI**
SMI → SB → SL	−0.192	0.268	[−0.735, 0.361]
SMI → SX → SL	−0.311	0.268	[−0.967, 0.064]
SMI → SB → SX → SL	0.309	0.199	[0.022, 0.777]

*Confidence intervals and standard errors are based on 10,000 bootstrap-samples (percentile bootstrap confidence intervals). n = 82. SMI, severity of the mental illness; SB, symptomatic burden; SX, social exclusion; SL, sick leave; SE_b_, standard error of the regression coefficient of the indirect effect; CI, confidence interval. Unstandardized regression coefficients*.

### Additional Analysis

Since the acquisition of patients for the present paper lasted until May 2020, a part of the participants undertook their assessment during the beginning of the COVID-19 pandemic in Germany. To rule out the possibility that the far-reaching influences of the pandemic on the society did affect the presented results we excluded any patient having participated in the year 2020 and reran the analyses, yielding similar results (c.f. Appendix A of the Supplementary Material).

Furthermore, some patients completed the questionnaire of “COMPARE work” (i.e., the questions regarding social exclusion and sick leave) before answering the questionnaire of “COMPARE family” (i.e., the questions regarding the symptomatic burden) resulting in a mixed temporal precedence. Thus, we excluded every participant who answered to “COMPARE work” one or more days before answering to “COMPARE family”, which resulted in patients completing the questionnaire of “COMPARE work” 12 days after the questionnaire of “COMPARE family”, on average. Afterwards we reran the analyses and obtained similar results (c.f. Appendix B of the Supplementary Material).

## Discussion

The goal of the present study was to contribute to the scarce literature on employees with a mental illness (EMI) by investigating the impact of the mental illness on sick leave. More specifically, we hypothesized and tested whether the severity of the mental illness has an indirect effect on the days of sick leave sequentially via the symptomatic burden and social exclusion at the workplace. Regression and path analysis supported our hypothesis, indicating that patients with a more severe mental disorder suffer from a stronger symptomatic burden. This in turn increases their experienced exclusion at the workplace, yielding an indirect effect of the severity of the mental illness on social exclusion. Finally, the isolation EMI experience at the workplace leads to an increase of absenteeism, resulting in the hypothesized serial mediation.

Although stigmatization and discrimination of people with a mental illness in general has been the scope of several investigations [for reviews on the topic see e.g., (21, 75, 76)], the work situation of EMI still is understudied (16). Previous investigations indicate that social distancing from people with a mental illness exists – not only in the general public but also in the work context [e.g., (21, 24)]. The resulting isolation is known to have detrimental effects on health [e.g., (27–29)] and thus, social exclusion can contribute to sick leave (30). The present study is the first that connects those findings, demonstrating that EMI show higher rates of absenteeism due to the social exclusion they experience at the workplace. It therefore adds to a better understanding of the experiences people with a mental illness make at the workplace and the consequences evoked thereof.

Deeper insights on the work situation of EMI are necessary since many of the published investigations on this population are descriptive in nature, limiting the possible conclusions drawn from those findings [c.f. (16)]. Thus, the current paper contributes to the scarce literature by applying inferential statistics, allowing more reliable conclusions about the specific work environment of EMI.

The study's findings underline the harmfulness of discrimination in general and social exclusion in particular. Various studies indicate the deleterious effects isolation can have on human health (27–29)—thus it is not surprising that socially excluded employees have a higher risk for a long-term sick leave spell (30). The present study replicates those findings in the population of employees with a mental illness, indicating that the social exclusion of EMI increases the days they are sick leaving. Besides the detrimental effects on the health of the discriminated individual, those results also imply negative consequences for organizations and the society as a whole given the amount of costs employers and states across the European Union spend in relation to absenteeism (77).

Although discriminative actions as social distancing toward EMI have been the scope of investigations before [e.g., (23)], this study is the first to portend that the symptomatic burden caused by the mental illness is crucial for the degree of exclusion EMI experience at their workplaces. Seen through the lens of the stereotype content model (SCM) (31) this finding makes sense: The model and its extension, the BIAS Map (32), propose that groups who are evaluated as being cold and incompetent as e.g., people with a mental illness (37), elicit active and passive harm (e.g., exclusion) (31–33). Thus, employees who are perceived as having more mental health problems should experience more social exclusion. However, to evaluate that a certain coworker has a mental health problem, the colleagues first have to notice corresponding peculiar behavior, that is, symptoms of a mental disorder. The more symptoms the colleagues notice (or the stronger they are), the higher the attributed mental health problems of the coworker and hence the elicited behavioral reaction will be. Thus, EMI suffering from a high symptomatic burden will be perceived as being more mentally ill by their coworkers in comparison to EMI with a low symptomatic burden, and thus experience more social exclusion. Experimental evidence supports the suggested importance of the symptomatic burden: Muschalla et al. (78) were able to show that the announced willingness of a fictious coworker with a mental illness to work on her mental health problems led to a lower desire for social distance toward that fictious coworker. Thus, even the anticipation of lower symptoms seems to mitigate the desire to socially distance oneself from the EMI.

### Limitations

The present paper has several limitations. First of all, cross-sectional data assessed at one point of time cannot be interpreted causally, since the temporal precedence remains unclear [c.f. (79)]. In the present study however, this concern can partly be thwarted: Although the variables belong to the same assessment, they have not all been measured at the same time. The predictor (severity of the mental disorder) has been rated by trained study personnel in the first interview session which always took part before the online-questionnaires were dispensed [c.f. (62)]. Furthermore, we found similar results when we reanalyzed the data without participants who answered to the questionnaire of “COMPARE work” one or more days before answering the questionnaire of “COMPARE family,” yielding an average of 12 days between the assessment of the first mediator (the symptomatic burden) and the second mediator/the outcome (social exclusion and sick leave). Thus, temporal precedence could at least partly be established.

Despite these efforts to ensure temporal precedence, the results still have to be interpreted with caution: Different investigations suggest that experienced exclusion is not only a consequence of a mental illness but also may contribute to its development [c.f. (80, 81)], making a reversed causation of the presented results possible. Furthermore, mental illnesses go along with an increased risk for physical disorders (82, 83). Thus, it is possible that comorbid physical impairments might have contributed to social exclusion and sick leave as well, confounding the results of the present study. Therefore, future replications applying a cross-lagged panel design and incorporating possible confounders are recommended to gain more certainty about the direction and validity of the presented effects [c.f. for instance (84)].

As in other clinical trials (85–87) recruitment of patients was difficult, yielding a small sample size in the present paper which bears the risk of a non-representative sample [c.f. for instance (84)]. In the German population, the most prevalent groups of mental disorders are anxiety disorders followed by affective disorders (88, 89). Similar results are found in non-German representative studies on mental illnesses in the working population, showing that simple phobia is the most prevalent mental disorder among the workforce followed by depression [c.f. (10)]. Although the present sample also mostly consists of patients with affective or anxiety disorders, the former are clearly dominating (40 diagnoses of affective disorders vs. 20 diagnoses of anxiety disorders). Thus, our sample is not an optimal representation of the population of employees with a mental illness. Future investigations applying a net online-questionnaire, including online screening instruments instead of on-site clinical interviews, could be able to establish a better representation of EMI by lowering the effort for participation.

Besides restrictions in the representativeness, the small sample size also constitutes a problem for the conducted analyses: Complex path models often require more participants than investigated in the present paper [c.f. (90)]. Unfortunately, although recruitment maintained for 17 months, it was not possible to acquire a larger sample—a problem different clinical trials are confronted with [c.f. (85–87)]. Since previous literature already provided support for the individual paths of the mediation model, indicating that a higher severity of the mental illness is associated with a higher symptomatic burden (42–45), that mental illnesses go along with social exclusion [e.g., (20, 21, 24, 38)] and that social exclusion in turn increases the risk for sick leave (30), we are confident that the depicted mediation model represents not merely a chance finding. However, future investigations should try to replicate our results within a larger sample.

Multiple sources of common method biases can operate in any given study and limit the trustworthiness of the results [c.f. (91)]. In the present paper we tried to confine this problem by several ways: First of all, we were able to draw on different assessment-methods (interviews and questionnaires), which might delimitate the risk of a common method bias to some degree [c.f. (91)]. Furthermore, the problem could partly be mitigated by the different contexts some of the variables were assessed in (more specifically, the different questionnaires of “COMPARE family” and “COMPARE work”) [c.f. (91)]. At last, the response format used to assess sick leave has been different from the response formats of its predictors which might reduce the risk of common method bias as well (91).

Besides actually being ill, employees may also call in sick for other non-health related reasons, as for instance caring for their ill children. To assure that we only measure the time employees were sick leaving because of their ill health, we explicitly asked for the days they were absent from work due to health reasons and appealed to exclude any days they were missing due to an illness of their children.

Lastly, the majority of the participants were female (77.9%). This can be explained by the applied inclusion criteria, only allowing people to participate if they fulfilled the criteria for a mental disorder [c.f. (62)]. In Germany, however, common mental illnesses as affective disorders or anxiety disorders have a higher prevalence in women than in men [c.f. (88, 89)]. Thus, the reason for the predominantly female composition of the sample probably lies in the applied inclusion criteria and mirrors the gender differences in the prevalence of common mental illnesses in Germany.

### Practical Implications and Future Research

The results of the present study indicate that the social exclusion of EMI can increase the days they are sick leaving. Thus, not only active forms of discrimination, which are [according to the extension of the SCM (31, 32)] executed with the blatant aim to affect the target group (e.g., bullying), have detrimental effects on the individual [e.g., (60, 92)], but also more passive forms that are marked by less directed effort as e.g., neglecting, ignoring or excluding [c.f. (32)], can affect individuals health and therefore also the organization they work in, e.g., due to indirect costs associated with absenteeism (15). While active forms of discrimination might at first view seem to be more threatening for the organizational health and probably—due to their more directed effort (32)—attract more attention in organizations, it is hence also necessary that supervisors pay attention whether the more passive forms of discrimination take place in their teams and, if so, try to counteract them.

One possibility to mitigate the social exclusion of EMI could be the appliance of workplace interventions. The Mental Health First Aid training (93, 94) for instance can increase the intentions to provide help to a person with mental health problems in general and might also reduce the desire for social distance, as a recent meta-analysis across several settings indicates (95). Although not specifically developed for the workplace (94), a recent randomized controlled trial shows that the training may also increase the willingness to help a fictious coworker with a mental illness among public servants (96). Future randomized controlled trials might investigate whether the training is able to prevent the detrimental process described in this paper, e.g., by reducing the social exclusion EMI experience in the workplace.

Practitioners, responsible for occupational health policies in their organization, should furthermore focus on reducing stigma toward EMI, since the stereotype, that people suffering from a mental illness are cold and incompetent, is responsible for their exclusion, as the SCM proposes [c.f. (31–33)]. According to a recent meta-analysis, the most promising approach to reduce stigma toward people with a severe mental disorder is establish contact and education [c.f. (97)]. Thus, practitioners can implement existing workplace programs as “The Working Mind” (98), which entails elements of education and contact (98, 99) and has proven to reduce mental health stigma (99), in order to counteract social exclusion of EMI at the workplace.

While the group “people with a mental illness” as a whole is perceived as being cold and incompetent in the sense of the SCM, research indicates that the perception on the two SCM-dimensions warmth and competence varies across different psychological disorders (23, 37). Follmer and Jones (23) for instance let participants rate how warm and competent different disorders are perceived in the workplace by society and found that individuals suffering from an anxiety disorder are perceived as being warmer and more competent than people with a depression or a bipolar disorder. As a result, the evoked behavioral response toward people with a mental illness might differ, depending on the disorder [c.f. (32)]: Employers for instance would rather dismiss an employee developing a schizophrenia than an employee developing a depression (17). Thus, it would be interesting to explore whether the presented results vary across different mental illnesses, more specifically, whether the type of disorder (for instance depression vs. anxiety disorder) moderates the effect of the symptomatic burden on social exclusion in the demonstrated process. Yet, when we checked our data, we were not able to find a significant interaction (c.f. Appendix C of the Supplementary Material), which might be caused by the small sample size. However, a better understanding of which subgroups of people with a mental illness are at special risk for social exclusion and the subsequent absenteeism at the workplace would help, e.g.„ for constructing more precise interventions, which is why we encourage further investigations in this regard.

## Conclusion

In 2016, about every sixth person in the European Union suffered from mental health problems. Besides the detrimental effects that go along with the disorder itself, people with a mental illness also face various hindrances imposed by society, that are known to worsen the overall health status. The present paper demonstrated that those effects also take place within the workforce. Employers therefore need to implement an integrative climate where employees with a mental illness can feel as safe and valued as every other employee – for the sake of their staff's but also their organization's well-being.

## Data Availability Statement

Data share of all primary data of the COMPARE-consortium is planed after the completion of the projects [see (61)]. Participants were also assured that data would not be shared until the projects were completed. Therefore, since assessments and projects are not yet completed, the data cannot yet be made available.

## Ethics Statement

The studies involving human participants were reviewed and approved by the Ethics Committee at the department of psychology at the Philipps-University Marburg. The patients/participants provided their written informed consent to participate in this study.

## Members of the COMPARE-Family Research Group

Kristin Gilbert, Department of Clinical Child and Adolescent Psychology, Faculty of Psychology, Philipps University Marburg, Marburg, Germany; Markus Stracke, Department of Clinical Child and Adolescent Psychology, Faculty of Psychology, Philipps University Marburg, Marburg, Germany; Christina Klose, Department of Medical Biometry, Institute of Medical Biometry and Informatics, Heidelberg University, Heidelberg, Germany; Johannes Krisam, Department of Medical Biometry, Institute of Medical Biometry and Informatics, Heidelberg University, Heidelberg, Germany; Moritz Pohl, Department of Medical Biometry, Institute of Medical Biometry and Informatics, Heidelberg University, Heidelberg, Germany; Claudia Buntrock, Department of Psychology, Chair for Clinical Psychology and Psychotherapy, Friedrich-Alexander University Erlangen-Nüremberg, Erlangen, Germany; David Daniel Ebert, Department of Psychology and Digital Mental Health Care, Faculty of Sports and Health Science, Technical University München, Munich, Germany; Jürgen Margraf, Department of Clinical Psychology and Psychotherapy, Faculty of Psychology, Ruhr-University Bochum, Bochum, Germany; Silvia Schneider, Department of Clinical Child and Adolescent Psychology, Faculty of Psychology, Ruhr-University Bochum, Bochum, Germany; Rudolf Stark, Department of Psychotherapy and Systems Neuroscience, Faculty of Psychology and Sports Science, Justus-Liebig-University Gießen, Gießen, Germany; Julia Metzger, Department of Psychotherapy and Systems Neuroscience, Faculty of Psychology and Sports Science, Justus-Liebig-University Gießen, Gießen, Germany; Julia Glombiewski, Department of Biopsychology, Clinical Psychology and Psychotherapy, Faculty of Psycholgy, University Koblenz-Landau, Landau, Germany; Anette Schröder, Department of Biopsychology, Clinical Psychology and Psychotherapy, Faculty of Psycholgy, University Koblenz-Landau, Landau, Germany; Jens Heider, Department of Biopsychology, Clinical Psychology and Psychotherapy, Faculty of Psycholgy, University Koblenz-Landau, Landau, Germany; Winfried Rief, Department of Clinical Psychology and Psychotherapy, Faculty of Psychology, Philipps University Marburg, Marburg, Germany; Pia Eitenmüller, Department of Clinical Psychology and Psychotherapy, Faculty of Psychology, Philipps University Marburg, Marburg, Germany.

## Author Contributions

BF, CT, KO, and NB generated the idea for the present paper. BF and CT performed the statistical analysis. BF wrote the first draft of the manuscript. CT contributed to sections of the manuscript. CR, CS, HC, KO, LW, MK, and RS designed the COMPARE-Consortium from which the used data originate and thus substantially contributed to the design of the present study. BF and NB were responsible for managing the project COMPARE work. The COMPARE-family research group was responsible for managing the project COMPARE family, for the clinical study monitoring as well as the data acquisition and data management of both projects. All authors revised the manuscript and approved the submitted version.

## Funding

The projects COMPARE work (grant number: 01GL1748A) and COMPARE family (grant number: 01GL1748B), from which the data used in this study originate, are entirely funded by the Federal Ministry of Education and Research (BMBF).

## Conflict of Interest

The authors declare that the research was conducted in the absence of any commercial or financial relationships that could be construed as a potential conflict of interest.

## Publisher's Note

All claims expressed in this article are solely those of the authors and do not necessarily represent those of their affiliated organizations, or those of the publisher, the editors and the reviewers. Any product that may be evaluated in this article, or claim that may be made by its manufacturer, is not guaranteed or endorsed by the publisher.

## Footnotes

^1^In the present paper we used data from the clinical study “COMPARE family” [see (62)] and the subproject “COMPARE work” [see (61)]. Since the acquisition of participants in the clinical study is still ongoing, two partial data sets were provided in May 2020 and in February 2021 with the aim to facilitate in advance analysis. The partial data sets contain final, not changeable and completely refined data. In this present paper we used data from the first partial data set provided in May 2020.
