# Supplementary material for: Leave Me Alone With Your Symptoms! Social Exclusion at the Workplace Mediates the Relationship of Employee's Mental Illness and Sick Leave
Source: Front Public Health. 2022 Jul 28;10:892174. doi: 10.3389/fpubh.2022.892174 (PMC9366673; doi:10.3389/fpubh.2022.892174)
Supplement: Supplementary file 1 [file Data_Sheet_1.pdf]

Appendix A

Results for hypotheses one and two without any patient having participated in the year 2020 to rule out the possibility of an influence of the COVID-19 pandemic on the results

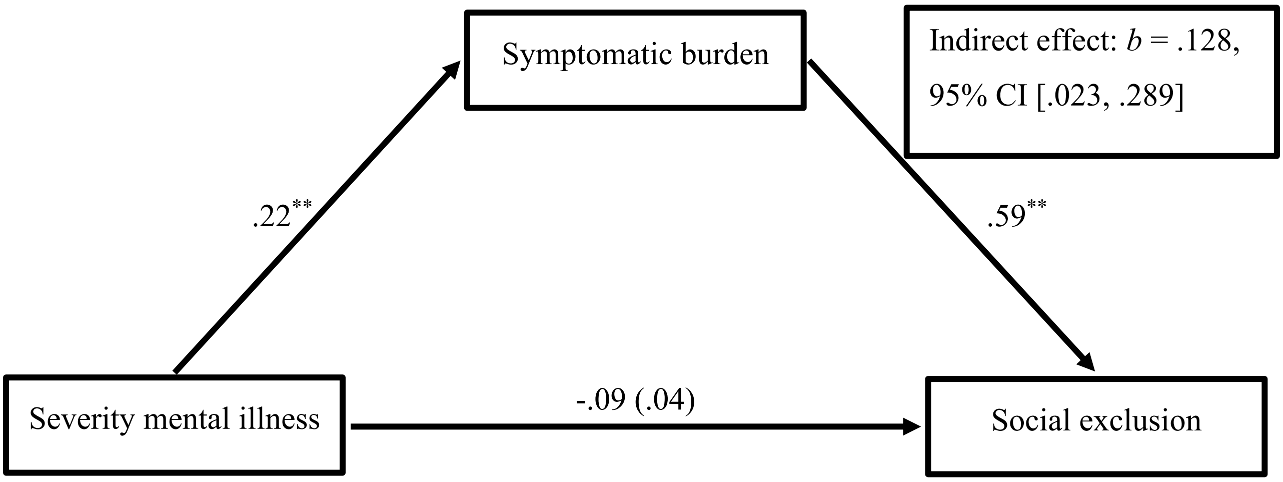

Figure A1. Mediation model showing the impact of the severity of the mental illness on social exclusion via the symptomatic burden (*n* = 70). The total effect of the severity of the mental illness on social exclusion without controlling for the symptomatic burden is shown in parentheses. Unstandardized regression coefficients. Two-sided testing of significance.
$^{**}p < .01$.

Results for hypotheses three without any patient having participated in the year 2020 to rule out the possibility of an influence of the COVID-19 pandemic on the results

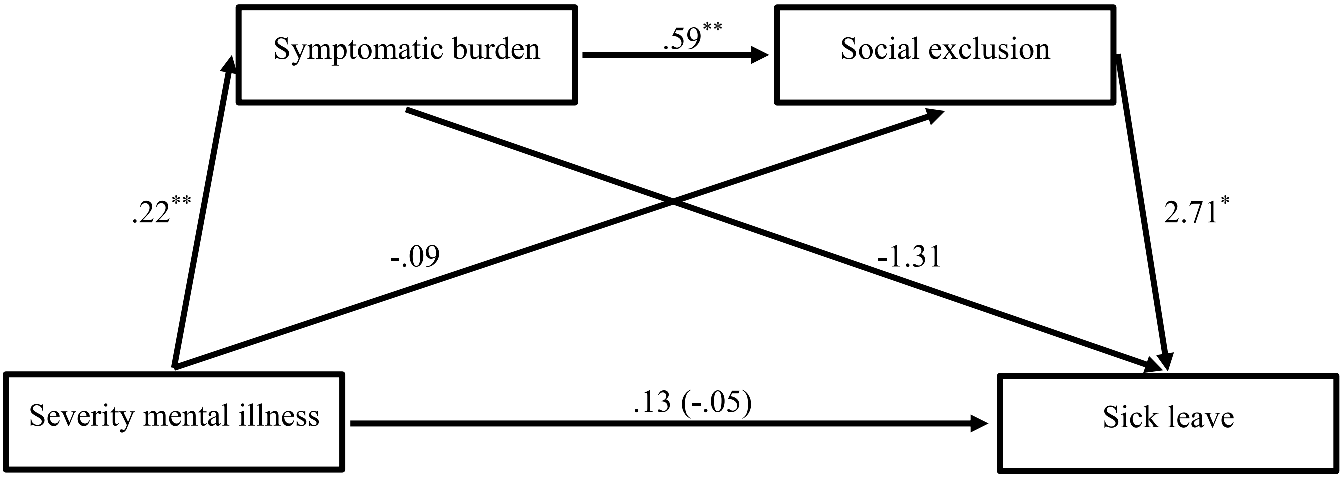

Figure A2. Mediation model showing the impact of the severity of the mental illness on sick leave via the symptomatic burden and social exclusion (n = 70). The total effect of the severity of the mental illness on sick leave without controlling for the symptomatic burden and social exclusion is shown in parentheses. Unstandardized regression coefficients. Two-sided testing of significance.
$^{*}p < .05$, $^{**}p <. 01$.

Table A3. *Indirect effects of the severity of the mental illness on sick leave.*

Effects	*b*	SE_b	95% CI
SMI → SB → SL	-.283	.359	[-.988, .471]
SMI → SX → SL	-.237	.284	[-.938, .193]
SMI → SB → SX → SL	.345	.232	[.006, .893]

Note. Confidence intervals and standard errors are based on 10,000 bootstrap-samples (percentile bootstrap confidence intervals). n = 70. SMI = severity of the mental illness; SB = symptomatic burden; SX = social exclusion; SL = sick leave; SE_b = standard error of the regression coefficient of the indirect effect; CI = confidence interval. Unstandardized regression coefficients.

Appendix B

Results for hypotheses one and two without any participant who answered to the questionnaire of compare work one or more days before answering to the questionnaire of compare family

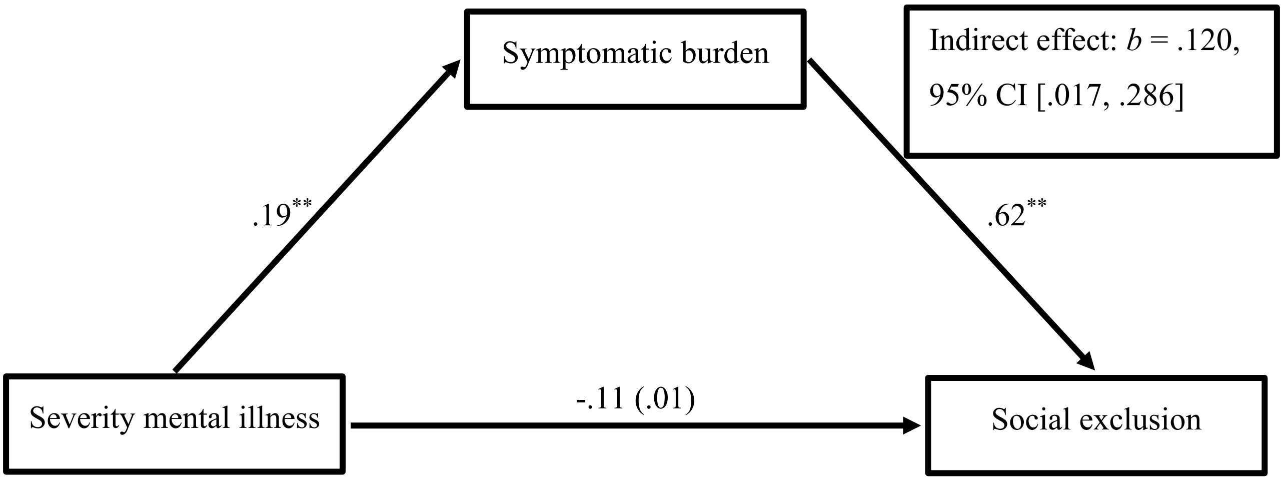

Figure B1. Mediation model showing the impact of the severity of the mental illness on social exclusion via the symptomatic burden (n = 66). The total effect of the severity of the mental illness on social exclusion without controlling for the symptomatic burden is shown in parentheses. Unstandardized regression coefficients. Two-sided testing of significance.
$^{**}p <. 01$.

Results for hypotheses three without any participant who answered to the questionnaire of compare work one or more days before answering to the questionnaire of compare family

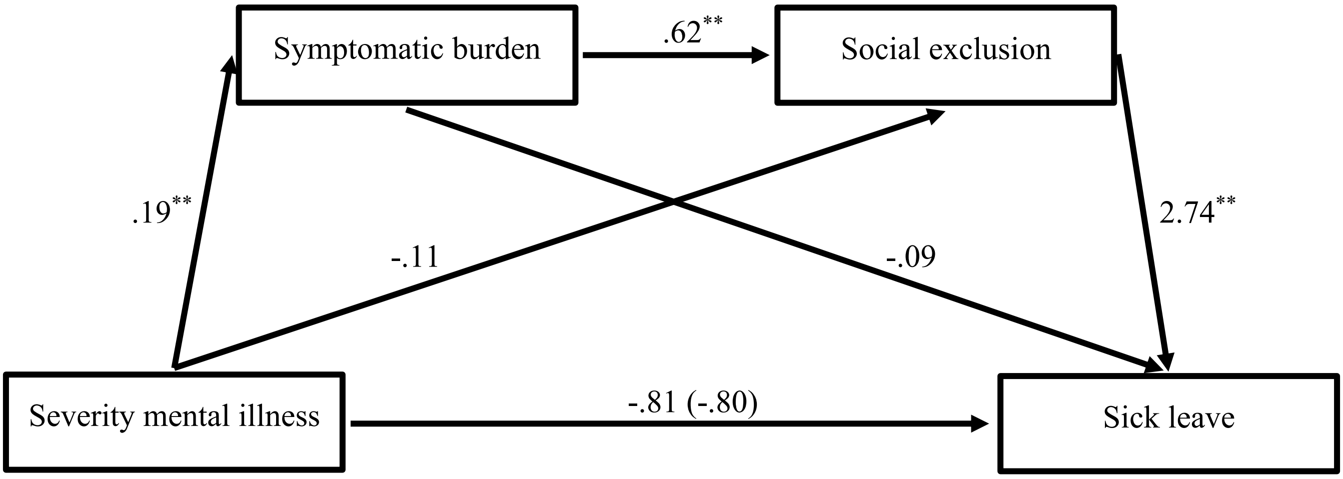

Figure B2. Mediation model showing the impact of the severity of the mental illness on sick leave via the symptomatic burden and social exclusion (n = 66). The total effect of the severity of the mental illness on sick leave without controlling for the symptomatic burden and social exclusion is shown in parentheses. Unstandardized regression coefficients. Two-sided testing of significance.
** p <. 01.

Table B3. *Indirect effects of the severity of the mental illness on sick leave.*

Effects	b	SE_b	95% CI
SMI → SB → SL	-.018	.254	[-.476, .542]
SMI → SX → SL	-.296	.327	[-1.083, .210]
SMI → SB → SX → SL	.329	.233	[.013, .900]

Note. Confidence intervals and standard errors are based on 10,000 bootstrap-samples (percentile bootstrap confidence intervals). n = 66. SMI = severity of the mental illness; SB = symptomatic burden; SX = social exclusion; SL = sick leave; SE_b = standard error of the regression coefficient of the indirect effect; CI = confidence interval. Unstandardized regression coefficients.

Appendix C

Results for a possible moderation of the relationship between the symptomatic burden and social exclusion by the type of disorder (depression vs. anxiety disorder)

Table C1. *Regression of social exclusion on symptomatic burden, disorder-type and their interaction.*

Predictors	*b*	SE_b	95% CI
Symptomatic burden	.558**	.196	[.164, .951]
Type of disorder [a]	.162	.227	[-.295, .619]
Symptomatic burden x Type of disorder	.367	.419	[-.475, 1.210]

Note. Symptomatic burden and type of disorder were mean centered prior to analysis. $n = 52$. SE_b = standard error of the regression coefficient; CI = confidence interval. Unstandardized regression coefficients. Two-sided testing of significance.
[a] 1= anxiety, 2 = depression.
** $p <. 01$.
